# *Prunus yedoensis* Bark Downregulates the Expression of Cell Adhesion Molecules in Human Endothelial Cell Lines and Relaxes Blood Vessels in Rat Aortic Rings

**DOI:** 10.3390/ph17070926

**Published:** 2024-07-10

**Authors:** Ye Eun Choi, Jung Mo Yang, Chae Won Jeong, Sujin Shin, Junkyu Park, Kyungjin Lee, Ju Hyun Cho

**Affiliations:** 1 Haram Central Research Institute, Cheongju 28160, Republic of Korea; ye0538@hanmail.net (Y.E.C.); ridely17@gmail.com (J.M.Y.); cjeong7979@gmail.com (C.W.J.); 2 Department of Korean Medicine, Graduate School, Kyung Hee University, Seoul 02447, Republic of Korea; sjshin04@khu.ac.kr; 3 Department of Science in Korean Medicine, Graduate School, Kyung Hee University, Seoul 02447, Republic of Korea; ojeoksan@khu.ac.kr; 4 Department of Herbal Pharmacology, College of Korean Medicine, Kyung Hee University, Seoul 02447, Republic of Korea; niceday@khu.ac.kr

**Keywords:** *Prunus yedoensis*, human endothelial cells, hypertension, vasorelaxation, cardiovascular disease

## Abstract

The incidence of cardiovascular diseases, such as high blood pressure, is increasing worldwide, owing to population aging and irregular lifestyle habits. Previous studies have reported the vasorelaxant effects of *Prunus yedoensis* bark methanol extract. However, various solvent extracts of *P. yedoensis* bark and their vascular relaxation mechanisms have not been sufficiently studied. We prepared extracts of *P. yedoensis* bark using various solvents (water, 30% ethanol, and 70% ethanol). *P. yedoensis* bark 30% ethanol extract (PYB-30E) decreased the expression of vascular cell adhesion molecule-1 (VCAM-1), intercellular adhesion molecule-1 (ICAM-1), and E-selectin in human umbilical vein endothelial cells (HUVECs) activated with 200 ng/mL TNF-α. Additionally, PYB-30E showed vasodilatory effects on isolated rat aortic rings. This was confirmed to be the result of the activation of the NO/cGMP pathway, regulation of non-selective calcium-activated K^+^ channels, and calcium channel blockade. Additionally, PYB-30E significantly reduced systolic and diastolic blood pressure in spontaneously hypertensive rats (SHR). Taken together, our results indicated that PYB-30E is a candidate functional material with preventive and therapeutic effects against hypertension.

## 1. Introduction

Cardiovascular diseases (CVDs) include hypertension, stroke, myocardial infarction, and angina, and are caused by heart and blood vessel disorders. Among them, hypertension causes disturbance of the endothelial system, vasoconstriction, and vascular hypertrophy [1,2]. The prevalence of hypertension is expected to increase due to the irregular lifestyles of modern society [3]. Recent research on the relationship between the onset of hypertension and cardiovascular disease and mortality indicates a need for early antihypertensive treatment and blood pressure management [4,5].

Smooth blood flow is important for maintaining body functions. Elevated blood pressure is a representative risk indicator of CVDs, and poor organization of vascular structures is a cause of CVDs [6]. The blood vessel wall is composed of several cells, including vascular smooth muscle cells (VSMCs), vascular endothelial cells (EC), and extracellular matrix (ECM). Nitric oxide (NO) produced in EC is known to affect vascular disorders by regulating vascular tension, inducing the relaxation of VSMC, and dilatation of vascular walls [7,8]. Blood pressure treatment with drugs (antihypertensive agents) is used to treat and prevent CVDs. These drugs include angiotensin-converting enzyme inhibitors, angiotensin receptor blockers, calcium-channel blockers, β-adrenergic blockers, and vasodilators [9,10]. Natural treatments using natural products may be an alternative.

*Prunus yedoensis* is a deciduous tree belonging to the *Rosaceae* family and is mainly distributed in East Asia, Korea, Japan, and the United States [11]. In traditional medicine, the bark of *P. yedoensis* has been used to treat cough, asthma, and urticaria, and has recently been reported to have antioxidant, anti-inflammatory, anti-diabetic, anti-allergic, anti-cancer, and liver-protective effects [12,13,14,15]. The major compound in *P. yedoensis* bark is prunetin, which has been reported to have various pharmacological effects, including anti-inflammatory, anti-obesity, and anti-osteoporotic effects [16,17,18,19]. Vascular relaxant and blood pressure regulatory effects of *Prunus* genus and prunetin in various areas have also been reported [20,21,22,23,24]. In particular, the methanolic extract of *P. yedoensis* bark shows a vasorelaxant effect by activating NO formation and blocking extracellular Ca^2+^ channels [25].

To further demonstrate the benefits of *P. yedoensis* bark for the development of pharmaceutical and functional foods, we prepared water, 30% ethanol, and 70% ethanol extracts of *P. yedoensis* bark. The effect of the extracts on the expression of vascular cell adhesion molecule-1 (VCAM-1), intercellular adhesion molecule-1 (ICAM-1), and E-selectin proteins in human umbilical vein endothelial cells (HUVECs) stimulated with TNF-α was investigated. Among them, the effects of *P. yedoensis* 30% extract (PYB-30E), which showed the best efficacy on endothelial cell activation, vascular relaxation, and blood pressure inhibition in the thoracic aortic ring of rats, were measured and the mechanism of action was evaluated.

## 2. Results

### 2.1. Effects of Prunus yedoensis Bark Extracts (PYB) and Prunetin on HUVECs Cell Viability

**Figure 1:**
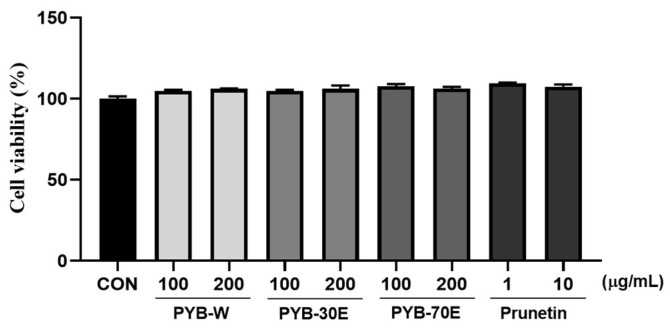
Effect of *Prunus yedoensis* bark extracts (PYB) and prunetin on the cell viability in human umbilical vein endothelial cells (HUVECs). The HUVECs were treated with PYB extracts of 100 and 200 μg/mL and prunetin from 1 to 10 μg/mL for 24 h. Cell viability was measured by MTT assay. Data were obtained from three independent experiments and are expressed as mean ± SEM. PYB-W, *P. yedoensis* water extract; PYE-30E, *P. yedoensis* 30% ethanol extract; PYE-70E, *P. yedoensis* 70% ethanol extract.

As shown in Figure 1, the cytotoxicity of PYB extracts in solvent and of prunetin on HUVECs was evaluated using the MTT assay after 24 h of treatment. The survival rate of HUVECs was not affected by PYB extracts at concentrations of 100 and 200 μg/mL, and prunetin at doses ranging from 1 to 10 μM.

### 2.2. Effects of PYB Extracts and Prunetin on TNF-α-Induced Human Vascular Endothelial Cell Activation

**Figure 2:**
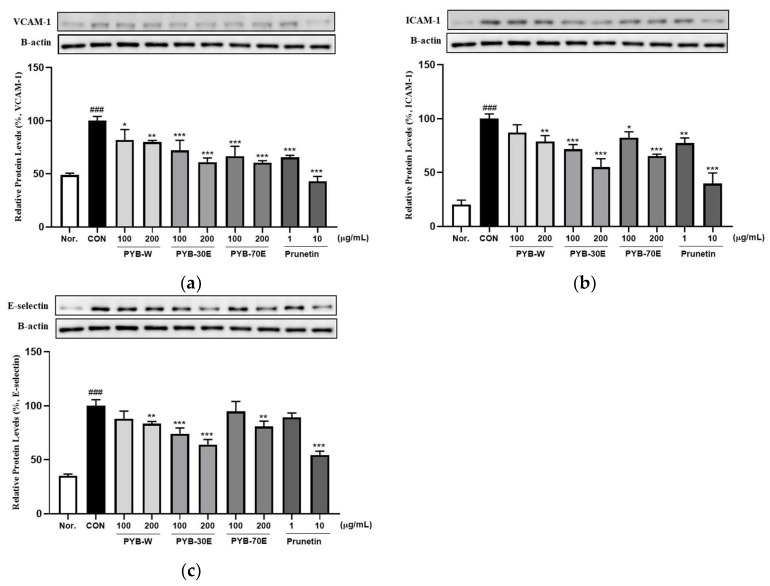
Effects of PYB extracts by solvent and prunetin on protein expression level of vascular cell adhesion molecule-1 (VCAM-1). (**a**) intercellular adhesion molecule-1 (ICAM-1), (**b**) E-selectin, (**c**) in HUVECs. HUVECs were pretreated with PYB of 100 and 200 μg/mL and prunetin from 1 to 10 μg/mL and TNF-α for 6 h. Protein expression levels were measured via western blot and analyzed using Image J software (1.8.0 version). Data were obtained from three independent experiments and are expressed as mean ± SEM. * *p* < 0.5, ** *p* < 0.01, *** *p* < 0.001 vs. CON. ### *p* < 0.001 vs. Nor. PYB-W, *P. yedoensis* water extract; PYE-30E, *P. yedoensis* 30% ethanol extract; PYE-70E, *P. yedoensis* 70% ethanol extract.

The overexpression of cell adhesion molecules (CAMs), including VCAM-1, ICAM-1, and E-selectin, in vascular endothelial cells is a phenotypic characteristic of endothelial dysfunction. It has been reported to be an early indicator of the development of cardiovascular diseases [26,27]. Western blotting was performed to investigate the protein expression of VCAM-1, ICAM-1, and E-selectin released from HUVECs cells activated with TNF-α. As shown in Figure 2, incubation with 200 ng/mL of TNF-α for 6 h significantly increased the expression of VCAM-1, ICAM-1, and E-selectin compared to the negative control group (not treated with TNF-α). However, there was a significant decrease in the expression of VCAM-1 after treatment with 100 and 200 μg/mL of PYB-W, PYB-30E, and PYB-70E. When treated with prunetin, which is the indicator component of PYB extracts, at concentrations 1 and 10 μg/mL, there was a significant decrease. There was a significant decrease in expression of ICAM-1 after treatment with 200 μg/mL of PYB-W, 100 and 200 μg/mL of PYB-30E, and PYB-70E. A significant decrease was also observed after treatment with 1 and 10 μg/mL prunetin. There was a significant decrease in expression of E-selectin after treatment with 200 μg/mL of PYB-W, PYB-70E and 100, 200 μg/mL PYB-30E. A significant decrease was observed after treatment with 10 μg/mL of prunetin. These results suggest that the PYB extracts and prunetin attenuate vascular endothelial cell activation by reducing CAMs induced by cytokines. As PYB-30E showed the greatest efficacy in regulating vascular endothelial cell activation, its vasorelaxant efficacy and its mechanism of action were investigated in rat thoracic aortic rings.

### 2.3. Effect of PYB-30E on Vascular Relaxation Depending on the Presence or Absence of Vascular Endothelial Cells

**Figure 3:**
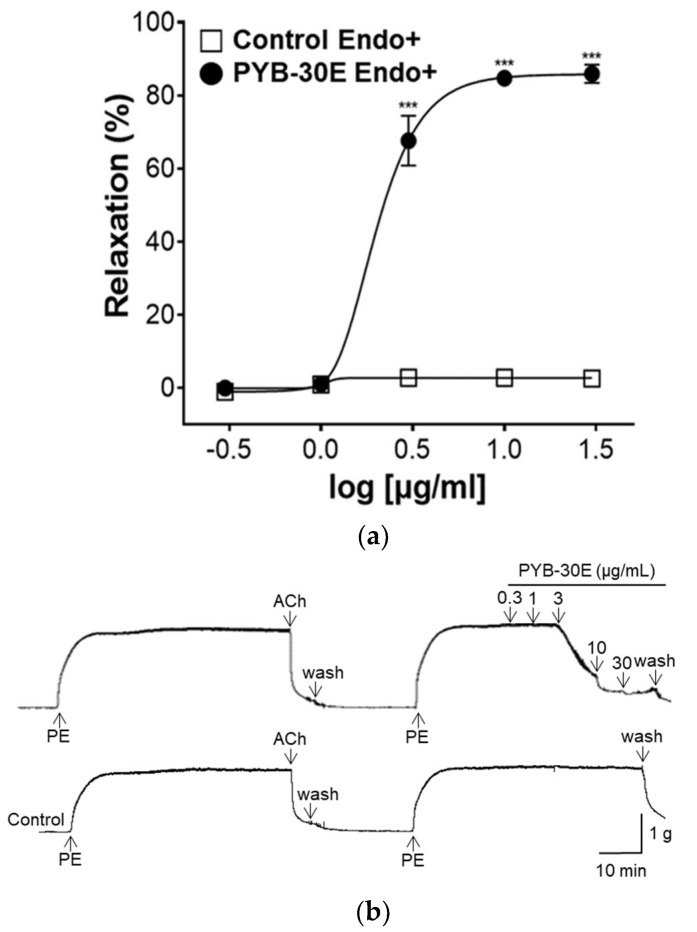

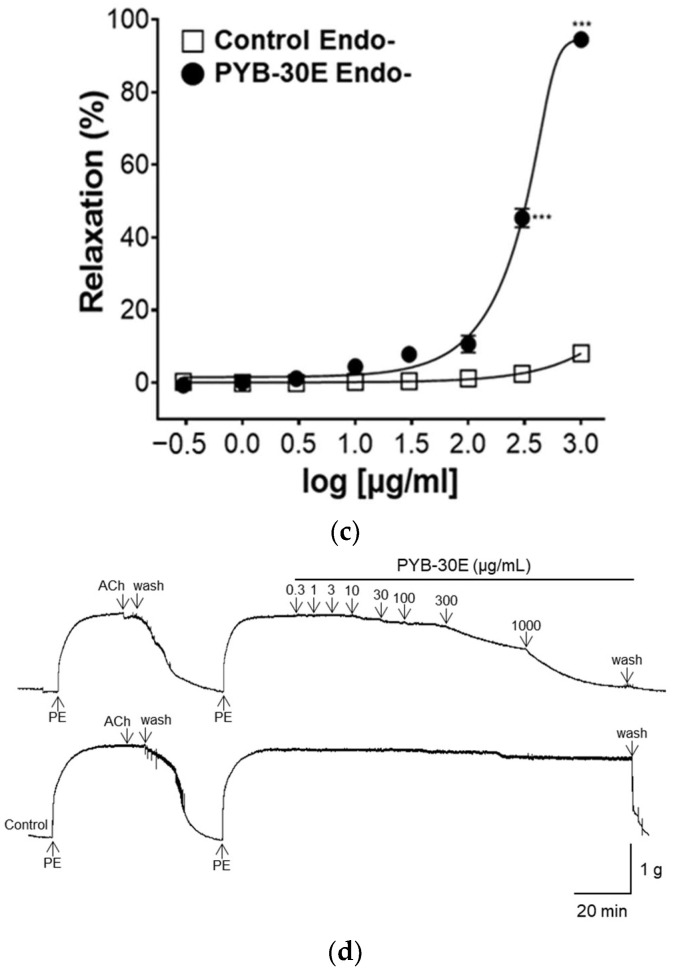
The relaxant effect of PYB-30E on PE (1 μM)-induced aortic ring contraction in vascular endothelium-intact [(Endo+)] (**a**), or -denuded [(Endo-)] rings (**c**). Representative trace under the indicated conditions (**b**,**d**). Data points represent mean ± SEM (*n* = 4). *** *p* < 0.001 vs. control. PE, Phenylephrine; Ach, Acetylcholine; PYB-30E, *P. yedoensis* 30% ethanol extract.

Vascular endothelial cells play an important role in regulating blood fluidity and vascular tone, including in the expansion and contraction of blood vessels, vascular smooth muscle cell proliferation, blood coagulation, and thrombosis. Damage to vascular endothelial cells causes blood clot formation and decreased elasticity of blood vessels, resulting in blood circulation disorders [28,29]. To determine whether the vasorelaxant property of PYB-30E stems from its effects on vascular endothelial cells, we used aortic rings constricted with phenylephrine (PE) and compared the vasorelaxant effects with and without vascular endothelial cells. When the plateau was attained, PYB-30E was added cumulatively (0.3, 1, 3, 10, 30 μg/mL). As shown in Figure 3A,B, when the aortic rings—(Endo+) with intact endothelial cells—were treated with PYB-30E at 0.3, 1, 3, 10 and 30 μg/mL, vascular relaxation rates were 0.07 ± 0.85, 1.05 ± 0.63, 67.59 ± 6.84, 84.72 ± 2.17, and 85.91 ± 2.53%, respectively. On the other hand, as shown in Figure 3C,D, when endothelial cell-free aortic rings (Endo-) were treated with PYB-30E at 0.3, 1, 3, 10, 30, 100, 300 and 1000 μg/mL, vascular relaxation rates were 0.64 ± 0.45, 0.13 ± 0.13, 1.12 ± 0.64, 4.42 ± 1.24, 7.83 ± 2.13, 10.66 ± 2.33, 45.35 ± 2.55, and 94.53 ± 0.64%, respectively. This indicates that low concentrations of PYB-30E promoted vascular relaxation by acting on endothelial cells.

### 2.4. Effect of PYB-30E on the Vasorelaxant Effect of Endothelium-Intact Aortic Rings Precultured with N ω-Nitro-L-arginine Methyl Ester (L-NAME), Indomethacin, 1-H-[1,2,4]-Oxadiazolo-[4,3-α]-quinoxalin-1-one (ODQ), and Methylene Blue (MB)

**Figure 4:**
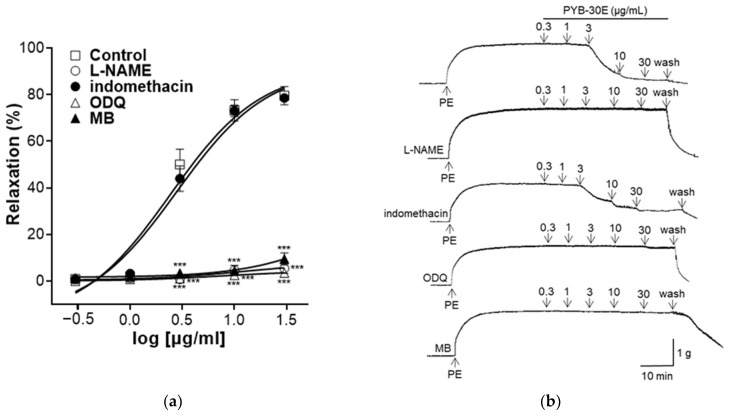
The relaxant effect of PYB-30E on PE (1 μM)-induced endothelial-intact aortic rings contraction in the presence or absence (Control) of L-NAME (100 μM), indomethacin (10 μM), ODQ (10 μM), and MB (10 μM). (**a**) Representative trace under the indicated conditions. (**b**) Data points represent mean ± SEM (n = 4–5). *** *p* < 0.001 vs. control. PE, Phenylephrine; L-NAME, NO synthase inhibitor; ODQ, 1-H-[1,2,4]-oxadiazolo[4,3-α]quinoxalin-1-one; MB, methylene blue. *** *p* < 0.001 vs. control.

The vascular endothelium contains endothelium-derived relaxing factors (EDRF) such as endothelium-derived hyperpolarizing factors (EDHF), prostacyclin (PGI_2_), and nitric oxide (NO) and endothelium-derived contracting factors (EDCF) such as endothelin-1, angiotensin II, and reactive oxygen species (ROS) on the endothelial surface. EDRF and EDCF regulate vascular relaxation and constriction, respectively, and endothelial dysfunction is characterized by a disturbance in the balance of EDRF and EDCF [30]. In endothelial cells, endothelial nitric oxide synthase (eNOS) activated by Ca^2+^/calmodulin binding produces NO, which increases the cellular concentration of cGMP in response to the NO concentration. The activation of the NO–cGMP pathway is a hallmark of vascular relaxation [31,32]. Additionally, the PGI_2_ vasorelaxant is produced by endothelial cells and the cyclooxygenase (COX) enzyme [33]. As shown in Figure 4, to confirm the vasorelaxation mechanism of PYB-30E on the vascular endothelium, the group was pretreated with L-NAME (NO synthase inhibitor), indomethacin (non-selective COX inhibitor), ODQ, and MB (sGC inhibitor), and compared with the non-pretreated group. When the plateau was attained, PYB-30E was added cumulatively (0.3, 1, 3, 10, 30 μg/mL). In the groups pretreated with L-NAME, ODQ, or MB, the vasorelaxant effects of PYB-30E were significantly suppressed. However, no significant differences were observed in the indomethacin-treated group. Clearly the vascular relaxation effect of PYB-30E was achieved through the NO-cGMP pathway, which is the main active pathway of endothelial cells, and its correlation with the PGI_2_ pathway was found to be minimal.

### 2.5. Effect of PYB-30E on Vasorelaxant Effect of Intact Endothelium Aortic Rings Pre-Incubated with Barium Chloride (BaCl_2_), 4-Aminopyridine (4-AP) and Tetraethylammonium Chloride (TEA)

**Figure 5:**
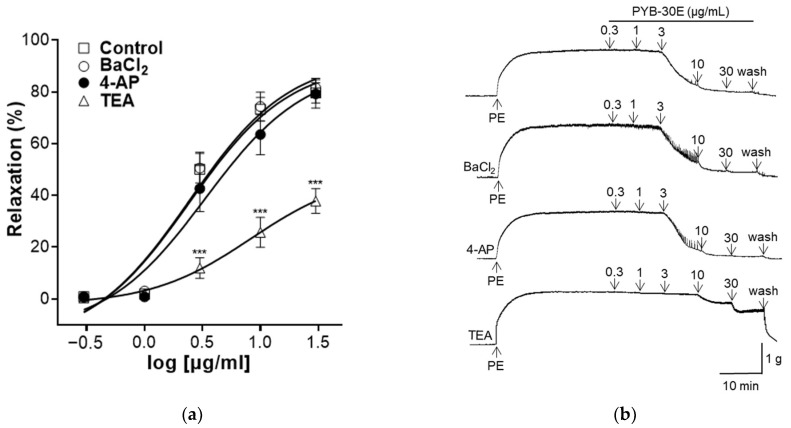
Effect of K^+^ channel blockers on PYB-30E-induced relaxation. The relaxant effect of PYB-30E on PE (1 μM)-induced contraction endothelial-intact aortic rings in the presence or absence (Control) of BaCl_2_ (10 μM), 4-AP (10 mM), TEA (1 mM). (**a**) Representative trace under the indicated conditions. (**b**) Data points represent mean ± SEM (n = 5–7). *** *p* < 0.001 vs. control. PE, Phenylephrine; BaCl_2_, Barium chloride; 4-AP, 4-aminopyridine; TEA, tetraethylammonium chloride.

K^+^ channels regulate the cell membrane potential of VSMCs and play an important role in regulating the contraction and relaxation of VSMCs [34]. Activation of K^+^ channels relax blood vessels by inhibiting the influx of Ca^2+^ through voltage-operated Ca^2+^ channels (VOCC) [35]. Representative types of K^+^ channels include inward-rectifier K^+^ channels, voltage-gated K^+^ channels (K_v_), and Ca^2+^-activated K^+^ channels (K_ca_) [36]. As shown in Figure 5, to evaluate the K^+^ channel-related mechanisms that regulate vascular contraction and relaxation in vascular smooth muscle, vascular sections were pretreated with each K^+^ channel blocker (BaCl_2_, 4-AP, TEA). When the plateau was attained, PYB-30E was added cumulatively (0.3, 1, 3, 10, 30 μg/mL). After treatment, the results were compared with those of the group that did not receive pretreatment. We found that the vasodilatory effect of PYB-30E was significantly suppressed in the group pretreated with TEA, and that there was no significant difference in the vasodilatory effect of PYB-30E when pretreated with BaCl_2_ and 4-AP. This suggests that the vasodilatory effect of PYB-30E is likely related to the regulation of potassium channels, specifically non-selective calcium-activated Ca^2+^ channels.

### 2.6. Effect of PYB-30E on Vasorelaxant Effect on Extracellular Ca^2+^-Induced Contraction

**Figure 6:**
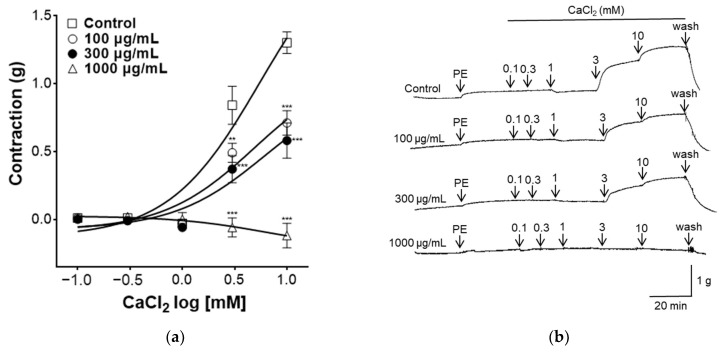
Effect of blocking Ca^2+^ channel PYB-30E-induced relaxation. The inhibitory effect of PYB-30E on CaCl_2_-induced contraction in endothelium-intact aortic rings pre-contracted by PE. (**a**) Representative trace under the indicated conditions. (**b**) Data points represent mean ± SEM (*n* = 4–5). ** *p* < 0.01, *** *p* < 0.001 vs. control. PE, Phenylephrine.

Increased intracellular Ca^2+^ concentration induces VSMC contraction. Ca^2+^ binds to the acidic protein calmodulin and phosphorylates the light chain of myosin, thereby inducing contraction of VSMCs [37]. As shown in Figure 6, to evaluate the effect of PYB-30E on Ca^2+^ influx, which regulates vascular contraction and relaxation in vascular smooth muscle, the concentration of CaCl_2_ was increased in the Ca^2+^-free K-H solution after the vascular sections were contracted with PE, and the vascular constriction was measured. When the plateau was attained, CaCl_2_ was added cumulatively (0.1, 0.3, 1, 3, 10 mM). The PYB-30E (100, 300 and 1000 μg/mL) pretreatment group showed a significant inhibitory effect on vasoconstriction compared with the control group. High concentrations of PYB-30E also exhibit vasorelaxant effects through a mechanism independent of endothelial cells (Figure 3C,D). Our results suggest that high concentrations of PYB-30E exert a vascular relaxant effect by blocking Ca^2+^ channels (Figure 6). The PYB-30E pretreatment group showed a significant inhibitory effect on vasoconstriction compared with the control group. This suggests that PYB-30E exerts a vascular relaxant effect by blocking calcium channels.

### 2.7. Effect of PYB-30E on Angiotensin II-Induced Vasoconstriction

**Figure 7:**
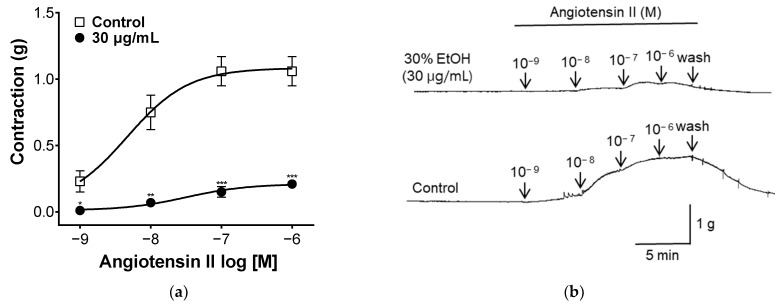
The Inhibitory effect of PYB-30E on endothelial-intact aortic rings constricted by angiotensin II (10^−9^, 10^−8^, 10^−7^, 10^−6^ M) (**a**). Representative trace under the indicated conditions (**b**). Data points represent mean ± SEM (*n* = 4–5). * *p* < 0.5, ** *p* < 0.01, *** *p* < 0.001 vs. control.

Angiotensin II is a hormone that affects vasoconstriction by binding to the Angiotensin II receptor type 1(AT1) receptor in vascular smooth muscle and increasing intracellular calcium concentrations [38]. To evaluate the angiotensin II-related mechanisms, blood vessel sections were treated with different concentrations of angiotensin II. As shown in Figure 7, when treated with angiotensin II, vascular segments contracted. Vascular constriction was suppressed in the group pretreated with PYB-30E. This suggests that PYB-30E may be taken to improve blood circulation disorders by inhibiting the action of angiotensin II.

### 2.8. Effect of PYB-30E on Lowering Blood Pressure in Spontaneously Hypertensive Rat (SHR)

**Figure 8:**
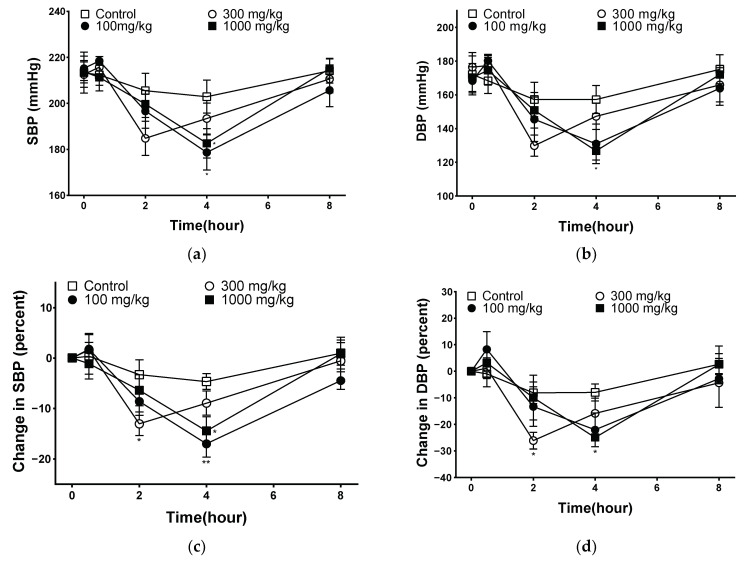
Blood pressure-lowering effect of PYB-30E in a spontaneously hypertensive rat (SHR). (**a**) Systolic blood pressure (SBP), (**b**) diastolic blood pressure (DBP), (**c**) percent changes in SBP, (**d**) percent changes in DBP. Data points represent mean ± SEM (*n* = 5–6). * *p* < 0.5, ** *p* < 0.01 vs. control.

Hypertension increases peripheral vascular resistance, which leads to a decrease in vascular elasticity and luminal diameter of the vasculature [39]. The goal of hypertension treatment is to maintain blood pressure at an appropriate level, and the measurement of systolic and diastolic blood pressures plays an important role in hypertension [40]. The blood pressure-lowering effect of PYB-30E was measured to evaluate improvements in blood circulation. As shown in Figure 8, systolic blood pressure (SBP) and diastolic blood pressure (DBP) were measured at 0 h before oral administration (baseline blood pressure), and 30 min, 2 h, 4 h, 8 h after the oral administration of PYB-30E to SHR. After 4 h, SBP decreased significantly in the 100 and 1000 mg/kg groups, and DBP decreased in the 1000 mg/kg group. The degree of change in blood pressure for each SHR due to differences in blood pressure between individual SHRs was calculated and compared with that of the control group. At 2 h after administration, the 300 mg/kg group showed a significant decrease in SBP and DBP. After 4 h of administration, SBP significantly decreased in the 100 and 1000 mg/kg groups, whereas DBP decreased in the 1000 mg/kg group. This suggests that PYB-30E lowers blood pressure and improves blood circulation by increasing blood vessel elasticity.

## 3. Discussion

The purpose of this study was to investigate the vascular relaxant effects of PYB and utilize it as a functional food ingredient for cardiovascular diseases. Previous studies reported that medicinal plants from the *Prunus* genus exhibited vasorelaxant effects [20,21,22,23]. In particular, the methanol extract of *P. yedoensis* bark relaxed blood vessels through activation of the NO-cGMP pathway and regulation of extracellular Ca^2+^ channels [25]. Previous studies have reported that prunetin is the main compound in *P. yedoensis* [41,42]. Prunetin exhibits an aortic ring relaxation effect in rats by blocking the calcium/potassium channel in the vascular smooth muscle. Its antihypertensive effect through blood pressure reduction in SHR has been reported [24]. We investigated in more detail the vasorelaxation mechanism of *P. yedoensis* bark as a functional food ingredient. We prepared extracts of *P. yedoensis* bark using water (PYB-W), 30% ethanol (PYB-30E), and 70% ethanol (PYB-70E). The effect of *P. yedoensis* extract (PYB) on the activation of vascular endothelial cells was evaluated, and its vasorelaxant effect and mechanism of action were evaluated in rat aortic rings.

The balance of endothelial cells plays a very important role in vascular diseases as endothelial cells regulate inflammation, thrombosis, vascular constriction, and dilation of blood vessels [43,44]. Activated endothelial cells exhibit upregulated surface expression of CAMs, including VCAM-1, ICAM-1, and E-selectin [45]. E-selectin mediates the migration of monocytes on the vascular endothelial surface, while VCAM-1 and ICAM-1 promote monocyte adhesion and migration through the vascular wall [46]. CAMs are also involved in vascular system-related inflammation, including coronary syndrome, hypertension, and endothelial cell activation [47,48]. This indicates that CAMs can be used as biomarkers for cardiovascular diseases. Cytotoxicity was measured by treating HUVECs with various concentrations of PYB and prunetin in each solvent. We found that 100 and 200 μg/mL of PYB and 1 and 10 μg/mL of prunetin were not cytotoxic. HUVECs were stimulated with 200 ng/mL TNF-α for 6 h and the expression of CAMs (VCAM-1, ICAM-1, E-selectin) released from activated endothelial cells was measured. The expression of VCAM-1, ICAM-1, and E-selectin increased in activated endothelial cells, and treatment with various concentrations of solvent-specific PYB and prunetin significantly decreased their expression. Among these, PYB-30E showed the highest reduction of CAMs. A previous study showed that higher phenol content and antioxidant activity were obtained when aqueous organic solvents compared to absolute organic solvents were used for plant extraction [49]. Among water, ethanol, and methanol extracts, 20% and 40% ethanol extracts showed more significant antioxidant potential and higher phenolic content [50]. In addition, polyphenolic compounds have been reported to improve cardiovascular remodeling and vascular function [51,52]. This suggests that PYB-30E may inhibit endothelial cell activation.

We measured the vasorelaxant effect and the mechanism of action of PYB-30E in the rat thoracic aorta. The aortic ring assay is a model for measuring vasomotor function and is used to assess vascular constriction and relaxation, blood pressure regulation, and molecular mechanisms in cardiovascular diseases including hypertension [53,54]. Vasoconstriction leads to endothelial dysfunction, characterized by increased peripheral vascular resistance, platelet hyperactivity, and endothelial cell damage. This impairs the normal function of the cardiovascular system [55,56]. Endothelial cells regulate vascular tone through the release of various vasodilator and constrictor factors, regulation of intracellular calcium concentration, and regulation of potassium channels in vascular smooth muscle cells [57]. We evaluated vasorelaxant effects in the presence or absence of vascular endothelial cells to determine whether PYB-30E acts on vascular endothelial cells. Low concentrations (3, 10, and 30 μg/mL) of PYB-30E exhibited vasorelaxant effects in rat thoracic aortic rings with intact endothelial cells. This indicates that endothelial cells play an important role in the vascular relaxant effect at low concentrations of PYB-30E.

Additionally, we evaluated the mechanism of action for the vasorelaxant effect of PYB-30E. The mechanism of vascular relaxation involves the action of the mediators EDCF, EDRF, and EDHF produced and released from the vascular endothelium, which regulate the relaxation and contraction of VSMCs. EDCF contains angiotensin II and ROS, whereas EDRF contains NO and PGI_2_ [58,59]. eNOS, a Ca^2+^/calmodulin-dependent enzyme, converts L-arginine into NO. NO diffuses into vascular smooth muscle cells causing a decrease in Ca^2+^ release from smooth muscle cells, thereby reducing smooth muscle cell contraction [60,61]. COX-2 converts arachidonic acid to prostaglandin H_2_ (PGH_2_), which is then converted into PGI_2_ by prostacyclin synthase [62,63]. PGI_2_ activates adenylyl cyclase (AC), leading to the synthesis of cAMP [64]. Ultimately, cAMP reduces the intracellular Ca^2+^ concentration by inhibiting Ca^2+^ influx into the cell and activating potassium channels to cause hyperpolarization [65,66].

In this study, PYB-30E showed a vasorelaxant effect in the group pretreated with the eNOS inhibitor L-NAME and the sGC inhibitors ODQ and MB. However, no vascular relaxation effect was observed in the group pretreated with indomethacin. This suggests that the vascular relaxant effect of PYB-30E is the result of activation of the NO/cGMP pathway, which is the main action pathway of endothelial cells.

Another mechanism that regulates vascular constriction and relaxation is the EDHF pathway, which activates Ca^2+^-dependent potassium channels in VSMCs [67]. Vasodilation promotes VSMC relaxation through the activation of K^+^ channels, increased extracellular K^+^, hyperpolarization of VSMCs, and inhibition of Ca^2+^ influx through VOCCs. Conversely, K^+^ closure promotes vasoconstriction by depolarizing VSMCs, opening VOCCs, and increasing Ca^2+^ influx [35,68]. K^+^ channels in VSMCs express several isoforms; the representative channels are K_v_ channels and inward-rectifying K^+^ channels (K_ir_ and K_ATP_) [69]. In this study, K^+^ channel-related mechanisms were evaluated by pretreatment with BaCl_2_, an inward rectifier potassium channel blocker; 4-AP, a voltage-gated K^+^ channel inhibitor; and TEA, a Ca^2+^-activated K^+^ channel blocker. PYB-30E showed a vasorelaxant effect in the group pretreated with TEA, indicating that it is related to the regulation of potassium channels, specifically non-selective Ca^2+^-activated K^+^ channels.

Increased intracellular Ca^2+^ concentrations promote vasoconstriction by stimulating myosin light chain kinase (MLCK) phosphorylation of the Ca^2+^-calmodulin complex [35,70]. In the present study, extracellular Ca^2+^-induced contractions were induced by adding CaCl_2_ to the aortic rings. Pretreatment with PYB-30E significantly inhibited vasoconstriction. This suggests that the vasorelaxant effects of PYB-30E are mediated via calcium channel blockade.

Angiotensin II, found in VSMCs, induces the production of inositol triphosphate (IP3) through the AT1 receptor. IP3 increased intracellular Ca^2+^ levels in the sarcoplasmic reticulum and caused VSMCs to contract [71]. In this study, the effect of PYB-30E on the angiotensin II mechanism was measured, and it was shown that pretreatment with PYB-30E inhibits the action of angiotensin II by inhibiting the vascular constriction caused by angiotensin II.

Hypertension and cardiovascular disease are associated with endothelial dysfunction, abnormal vasoconstriction, and vascular relaxation [72]. Lowering BP early has been shown to significantly reduce the risk of cardiovascular diseases, including hypertension [73,74]. To measure the blood pressure-lowering effect of PYB-30E, the SBP and DBP of SHRs were measured using a tail cuff. In this study, oral administration of PYB-30E resulted in a significant decrease in SBP and DBP 4 h after administration.

In our study, PYB-30E significantly attenuated the activation of endothelial cells and showed a vascular relaxation effect through regulation of the NO-cGMP pathway, K^+^ and Ca^2+^ channels, and, finally, a blood pressure-lowering effect in SHR. This suggests that PYB-30E could potentially be used as a functional food to prevent or treat cardiovascular diseases such as high blood pressure.

## 4. Materials and Methods

### 4.1. Chemicals

We purchased Prunetin (CAS registry# 552-59-0, ≥98.0% (TLC), cat# 82415) from Sigma Aldrich (ST. Louis, MO, USA). The F-12K media was purchased from ATCC (Manassas, VA, USA). Heparin, tumor necrosis factor-α (TNF-α), radioimmunoprecipitation assay (RIPA) buffer, acetylcholine (ACh), phenylephrine (PE), N ω-Nitro-L-arginine methyl ester (L-NAME), indomethacin, 1-H-[1,2,4]-oxadiazolo-[4,3-α]-quinoxalin-1-one (ODQ), methylene blue (MB), Barium chloride (BaCl_2_), 4-aminopyridine (4-AP), tetraethylammonium chloride (TEA), ethylene glycol-bis (2-aminoethylether)-N,N,N′,N′-tetra acetic acid (EGTA), calcium chloride (CaCl_2_), dimethyl sulfoxide (DMSO), and angiotensin II were purchased from Sigma Aldrich (ST. Louis, MO, USA). Penicillin/streptomycin (P/S), trypsin-ethylenediaminetetraacetic acid (trypsin-DETA, 0.05%), and fetal bovine serum were purchased from Gibco (Waltham, MA, USA). MTT assay kits were obtained from DoGenBio (Seoul, Republic of Korea). Human EC Growth supplement (ECGC) was purchased from Cell Application (Inc, San Diego, CA, USA). Bovine serum albumin (BSA) and enhanced chemiluminescence (ECL) kits were purchased from Bio-Rad (Hercules, CA, USA).

### 4.2. Sample Preparation

*Prunus yedoensis* bark was purchased from Dong-Yang Herb (Seoul, Republic of Korea) in 2022. Dried *P. yedoensis* bark was mixed with water, 30% ethanol, and 70% ethanol. The water extract was obtained by boiling at 120 °C for 4 h, and the 30% and 70% ethanol extracts were extracted at 95 °C for 4 h in a reflux device. After filtration with a 106 μm pore size test sieve (Chung Gye Inc., Seoul, Republic of Korea), the extracts were concentrated in a vacuum rotary evaporator (Eyela, Tokyo Rikakikai Co. Ltd., Tokyo, Japan). Subsequently, *P. yedoensis* bark extracts (PYB-W, PYB-30E, and PYB-70E) for each solvent were obtained by freeze-drying (Labcono, Kansas City, MO, USA). Prunetin has been reported as the main compound of *P. yedoensis* bark [41,42]. Prunetin was purchased from Sigma (Sigma Aldrich, ST. Louis, MO, USA) and was used by diluting it in DMSO.

### 4.3. Cell Culture

Human umbilical vein endothelial cells (HUVECs) were purchased from the American Type Culture Collection (ATCC, Manassas, VA, USA). For cell culture, F-12K media containing 10% Fetal Bovin Serum (FBS), 100 units/mL penicillin/streptomycin, 0.1 mg/mL heparin, and 0.05 mg/mL Human EC Growth supplement (ECGC) were used. The HUVECs were cultured in an incubator (Thermo Fisher Scientific, Waltham, MA, USA) in the presence of 5% CO_2_ at 37 °C.

### 4.4. Cell Viability

HUVECs were distributed at 1 × 10^5^ cells/mL in 12 well plates coated with 0.1% gelatin and cultured for 24 h until cell confluence. Different concentrations of PYB, prunetin and 200 ng/mL of TNF-α were used for the treatment, and incubated for 6 h. Cell viability was measured using a microplate reader (Epoch; Bitek Instruments, Lnc., Winooski, VT, USA) at a wavelength of 450 nm after replacing the medium with 10% MTT reagent and allowing the solution to react at 37 °C for 1 h.

### 4.5. Western Blot

To confirm the intracellular expression of vascular cell adhesion molecule-1 (VCAM-1), intercellular adhesion molecule-1 (ICAM-1), and E-selectin, 1 × 10^5^ cells were placed in a 12 well plate coated with 0.1% gelatin. The cells were cultured for more than 24 h until the cells were confluent. After treatment with 200 ng/mL of TNF-α and PYB at different concentrations, the cells were cultured for 6 h. Cultured cells were washed with PBS, harvested by adding RIPA buffer, and centrifuged. The supernatant was collected, and the protein concentration was quantified using a BSA kit. Proteins were separated by electrophoresis on a 10% sodium dodecyl sulfate (SDS)-polyacrylamide gel. The separated proteins were transferred to a polyvinylidene fluoride (PVDF) membrane at 100 V for 1 h, blocked in 5% skim milk, and washed three times for 15 min with Tris-buffered saline containing 5% Tween-20 (TBS-T). The reaction was performed with a primary antibody diluted 1:200–1:1000 and then washed with TBS-T. Next, horseradish peroxidase (HRP)-conjugated anti-rabbit IgG and anti-mouse IgG were diluted to 1:3000 and allowed to react for 1 h. The membranes were then washed with TBS-T. The level of protein expression was confirmed using the ChemiDoc™ imaging system (Bio-Rad, Hercules, CA, USA). The membranes were incubated using an ECL kit for light emission, and then imaged using an imaging densitometer (ImageJ bundled with 64-bit Java 1.8.0_112). The expression levels were quantified.

### 4.6. Animals and Experimental Design

Six- to seven-week-old Sprague–Dawley Rats weighing 220–260 g were purchased from Daehan Biolink (Daehan Biolink, Eumseong-gun, Republic of Korea) and 40-week-old spontaneously hypertensive rats (SHR) weighing 225–325 g were purchased from Japan SLC (SLC inc., Shizuoka, Japan). All the animal procedures were approved by the Animal Care and Use Committee. Animals were maintained in a controlled environment (12/12 h light/dark cycles, temperature at 23 ± 2 °C) and had access to food and water ad libitum.

### 4.7. Fabrication and Preparation of Rat Thoracic Aortic Rings

After anesthetizing Sprague–Dawley rats (6–7 weeks old) with urethane, the chests were immediately incised, and the thoracic aortas were removed. The extracted blood vessels were immediately supplied with a gas mixture of 95% O_2_ and 5% CO_2_ and maintained at 37 °C in Krebs–Henseleit solution (K–H solution, composition, mM: NaCl, 118.0; KCl, 4.7; MgSO_4_, 1.2; KH_2_PO_4_, 1.2; CaCl_2_, 2.5; NaHCO_3_, 25.0; and glucose, 11.1; pH 7.4). The tissue and fat around blood vessels were removed, and the vessels were cut into rings approximately 2 mm in length. In experiments requiring the removal of the endothelium, the lumen of the vessel was removed by scraping with a thin cotton swab. The lower and upper parts of the fabricated thoracic aortas were hung with stainless steel hooks, the lower parst were connected to hooks installed on the bottom of the organ bath, and the upper parts were connected to an isometric force transducer connected to a physiograph to measure changes in isotonic contraction using PowerLab Chart 7.0 software (ADI instrument Co., Sydney, NSW, Australia) program. The aortic segments were stabilized in an organ bath, loaded with 1 g of passive tension, and stabilized for 1 h prior to the next experiment.

### 4.8. Measurement of Vascular Relaxation for Endothelium-Intact and -Denuded Aortic Rings

To investigate the involvement of the vascular endothelium in the vascular relaxation mechanism of PYB-30E, the degree of vascular relaxation was measured in endothelium-intact and endothelium-denuded aortic rings. When aortic rings pre-contracted with phenylephrine (PE, 1 μM) were relaxed with acetylcholine (Ach, 10 μM), the presence of functional endothelium resulted in relaxation of more than 85%, whereas rings with the endothelium removed resulted in less than 10% relaxation. Vasorelaxant effects of 0.3, 1, 3, 10, and 30 μg/mL PYB-30E were measured with and without vascular endothelium.

### 4.9. Confirmation of Vascular Relaxation Mechanism of Vascular Endothelium

To investigate the effect of PYB-30E on the cGMP and PGI_2_ pathways, aortic rings were pretreated with NG-nitro-L-arginine methyl ester (L-NAME, NO synthase inhibitor, 100 μM), indomethacin (cyclooxygenase inhibitor, 10 μM), 1-H-[1,2,4]-oxadiazolo [4,3-α]quinoxalin-1-one (ODQ, soluble guanylate cyclase inhibitor, 10 μM), or methylene blue (MB, cGMP inhibitor, 10 μM) for 20 min. Afterwards, the aortic rings were contracted with PE (1 μM) and then treated with PYB-30E at different concentrations (0.3, 1, 3, 10, 30 μg/mL) to compare the vascular relaxation effect with the control group that was not treated with the inhibitors.

### 4.10. Measurements of Vascular Relaxation in Aortic Rings Treated with K^+^ Channel Blockers

To investigate the effect of PYB-30E on K^+^ channel-induced vascular relaxation, aortic rings were treated with barium chloride (BaCl_2_, inwardly rectifying K^+^ channel blocker, 10 μM), 4-aminopyridine (4-AP, voltage-dependent K^+^ channel blocker, 1 mM), or tetraethylammonium chloride (TEA, non-selective calcium-activated K^+^ channel blocker, 1 mM) for 20 min. Afterwards, the aortic rings were contracted with PE (1 μM) and then treated with PYB-30E at different concentrations (0.3, 1, 3, 10, 30 μg/mL) to compare the vascular relaxation effect with the control group that was not treated with the blockers.

### 4.11. Measurement of Vasoconstriction by Extracellular Ca^2+^-Induced Contraction

To investigate the vasorelaxant effect of PYB-30E on the calcium channel blockade, aortic rings were deprived of Ca^2+^ with ethyleneglycol-bis(2-aminoethylether)-N,N,N′,N′- tetraacetic acid (EGTA, 1 mM). After stabilization in K–H buffer, the aortic rings were pretreated with PYB-30E (100, 300, 1000 μg/mL) for 20 min. Afterwards, PE (1μM) was administered to the aortic rings, and 20 min later, CaCl_2_ (0.1, 0.3, 1, 3, 10 mM) was added to determine the degree of vasoconstriction induced by Ca^2+^ at different concentrations (100, 300, 1000 μg/mL) of PYB-30E, and compared to the untreated control.

### 4.12. Measurement of Vascular Relaxation Caused by Ang II Treatment

To investigate the vascular relaxation mechanism of PYB-30E due to angiotensin II receptor, aortic rings were pretreated with PYB-30E (30 μg/mL) for 20 min. The degree of vascular constriction after treatment with Angiotensin II (10^−9^, 10^−8^, 10^−7^, 10^−6^ M) was compared with that of the control group not treated with PYB-30E.

### 4.13. Measurement of Blood Pressure Drop in SHR

In SHR, systolic blood pressure (SBP) and diastolic blood pressure (DBP) were measured using a tail cuff (CODA 8-Channel) before treatment and at 30 min, 2 h, 4 h, and 8 h after administration of the extract. Measurements were performed using a high throughput noninvasive blood pressure system (Kent Scientific Co., Ltd., Torrington, CT, USA). Distilled water, for the control group, and PYB-30E (100, 300, and 1000 mg/kg) were orally administered to each group.

### 4.14. Statistical Analysis

All data are expressed as the mean ± standard error of the mean (SEM). All data were subjected to Dunnett’s and Bonferroni tests using GraphPad Prism 8 (GraphPad Software, San Diego, CA, USA). A student’s t-test was used for comparison between the two groups. The statistical significance was set at *p* < 0.05.

## 5. Conclusions

In summary, our study confirmed that PYB-30E attenuates endothelial cell activation by downregulating ICAM-1, VCAM-1, and E-selectin. In addition, PYB-30E showed a vasodilatory effect in the rat thoracic aortic rings, and it was confirmed that this was the result of the regulation of the NO-cAMP pathway and K^+^ and Ca^2+^ channels. Additionally, the SBP and DBP reduction effects of PYB-30E on SHRs were confirmed. Our results confirm the potential of *Prunus yedoensis* bark as a functional food material. Additional research is necessary, including safety and stability analyses, standardization using indicator compounds, and toxicity evaluation of the indicator compounds in PYB-30E.

## Data Availability

The original contributions presented in the study are included in the article material, further inquiries can be directed to the corresponding authors.
